# Molecular heterogeneity in human papillomavirus‐dependent and ‐independent vulvar carcinogenesis

**DOI:** 10.1002/cam4.1633

**Published:** 2018-07-20

**Authors:** Dorian R. A. Swarts, Quirinus J. M. Voorham, Annina P. van Splunter, Saskia M. Wilting, Daoud Sie, Divera Pronk, Marc van Beurden, Daniëlle A. M. Heideman, Peter J. F. Snijders, Chris J. L. M. Meijer, Renske D. M. Steenbergen, Maaike C. G. Bleeker

**Affiliations:** ^1^ Cancer Center Amsterdam Department of Pathology, VU University Medical Center VU University Medical Center Amsterdam The Netherlands; ^2^ Department of Gynecology Antoni van Leeuwenhoek Hospital Amsterdam The Netherlands; ^3^ Present address: Quirinus J. M. Voorham, Stichting PALGA Houten The Netherlands; ^4^ Present address: Saskia M. Wilting, Department of Medical Oncology Erasmus Medical Center Rotterdam The Netherlands; ^5^ Present address: Divera Pronk, Hartwig Medical Foundation Amsterdam The Netherlands

**Keywords:** copy number alterations, human papillomavirus, progression risk, vulvar intraepithelial neoplasia, vulvar squamous cell carcinoma

## Abstract

Vulvar squamous cell carcinoma (VSCC) and precancerous vulvar intraepithelial neoplasia (VIN) can develop through human papillomavirus (HPV)‐dependent and ‐independent pathways, indicating a heterogeneous disease. Only a minority of VIN progress, but current clinicopathological classifications are insufficient to predict the cancer risk. Here we analyzed copy number alterations (CNA) to assess the molecular heterogeneity of vulvar lesions in relation to HPV and cancer risk. HPV‐status and CNA by means of whole‐genome next‐generation shallow‐sequencing were assessed in VSCC and VIN. The latter included VIN of women with associated VSCC (VIN^VSCC^) and women who did not develop VSCC during follow‐up (VIN ^no^ ^VSCC^). HPV‐testing resulted in 41 HPV‐positive (16 VIN^VSCC^, 14 VIN ^no^ ^VSCC^, and 11 VSCC) and 24 HPV‐negative (11 VIN^VSCC^ and 13 VSCC) lesions. HPV‐positive and ‐negative VSCC showed a partially overlapping pattern of recurrent CNA, including frequent gains of 3q and 8q. In contrast, amplification of 11q13/cyclinD1 was exclusively found in HPV‐negative lesions. HPV‐negative VIN^VSCC^ had less CNA than HPV‐negative VSCC (*P *=* *.009), but shared chromosome 8 alterations. HPV‐positive VIN ^no^ ^VSCC^ had less CNA than VIN^VSCC^ (*P *=* *.022). Interestingly, 1pq gain was detected in 81% of HPV‐positive VIN^VSCC^ and only in 21% of VIN ^no^ ^VSCC^ (*P *=* *.001). In conclusion, HPV‐dependent and ‐independent vulvar carcinogenesis is characterized by distinct CNA patterns at the VIN stage, while more comparable patterns are present at the cancer stage. Cancer risk in VIN seems to be reflected by the extent of CNA, in particular chromosome 1 gain in HPV‐positive cases.

**WHAT'S NEW**

Given that vulvar (pre)cancerous lesions present as a heterogeneous disease with only a minority of vulvar intraepithelial neoplasia's (VIN) progressing to cancer, there is a clinical need to identify women with VIN at risk for cancer. Using whole‐genome shallow‐sequencing on HPV‐positive and HPV‐negative VIN and vulvar squamous cell carcinoma (VSCC), we were able to identify chromosome 1 gain as a strong indicator for cancer risk in HPV‐positive VIN.

## 1 INTRODUCTION

The development of vulvar squamous cell carcinomas (VSCC) is recognized to be heterogeneous.^1^ Approximately 30%‐40% of the VSCC are estimated to be attributable to infection with high‐risk human papillomavirus (HR‐HPV), while in the remaining cases inflammatory conditions like vulvar lichen sclerosus (LS) are risk factors for VSCC development.^2^, ^3^, ^4^, ^5^ High‐grade vulvar intraepithelial neoplasia (VIN) is considered the precursor lesion of VSCC and can be categorized into HPV‐associated usual VIN (uVIN) and HPV‐negative differentiated VIN (dVIN). However, in clinical practice, the categorization into uVIN or dVIN is only done in a minority of high‐grade VIN and HPV testing is only performed in a minority of VIN.^5^ Studies have shown that, in contrast to the majority of VSCC being HPV‐negative, the majority of high‐grade VIN is HPV‐positive.^3^, ^5^ Different factors might explain this paradoxical situation: (1) compared to HPV‐positive VIN, HPV‐negative VIN is both clinically and histopathologically less likely to be identified, and (2) HPV‐negative VIN appears to have a higher cancer progression risk of at least 30%‐35% with a shorter time interval between the precancerous VIN phase and invasive cancer.^6^, ^7^ For HPV‐positive VIN, the cancer progression risk has been estimated between 3% and 9% in treated patients and about 16% in untreated patients.^7^, ^8^, ^9^

Given that only a minority of VIN will progress to cancer and most affected women are treated similarly with often mutilating interventions,^10^, ^11^ there is a clinical need for objective biomarkers that can predict the cancer risk in VIN providing opportunities for risk‐guided tailored management of women affected with VIN. Molecular markers reflecting genetic and epigenetic host cell alterations associated with vulvar carcinogenesis are expected to be most promising. However, studies on genetic and epigenetic aberrations in VIN and VSCC are limited.^1^, ^2^, ^12^, ^13^, ^14^, ^15^, ^16^ *TP53* mutations are frequently (~30%) detected in HPV‐negative VSCC as well as HPV‐negative VIN (~21%), suggesting that *TP53* mutations might be involved at an early stage.^1^ Immunohistochemically HPV‐positive VIN shows usually absence of p53 and strong p16 expression.^2^ VSCC exhibit frequent gains of chromosome 3q and 8q, and losses of 3p, 8p, and 11q.^12^, ^14^, ^16^, ^17^, ^18^, ^19^, ^20^, ^21^

To obtain better insight in the molecular events underlying vulvar carcinogenesis, this study was set out to comprehensively analyze HPV‐status and DNA copy number alterations (CNA) in a well‐characterized series of VIN and VSCC. To allow identification of specific CNA that may reflect the cancer risk for women with VIN, CNA in VIN of women with VSCC were compared with VIN of women without VSCC.

## 2 MATERIALS & METHODS

### 2.1 Selection of vulvar lesions

Cases of VIN and VSCC were retrieved from the pathology archives of the Departments of Pathology of VU University Medical Center (n = 26), the Academic Medical Center (n = 25) and the Netherlands Cancer Institute/Antoni van Leeuwenhoek Hospital (n = 4), Amsterdam, The Netherlands. VIN adjacent to VSCC was considered to be a surrogate of the most advanced precancerous stage of VIN. Selected cases were enriched with p16 positive cases to achieve comparable group numbers as in regular care only a minority of VSCC cases is p16 (and HPV) positive. Selected cases were reviewed and tissue blocks were selected for sufficient VSCC and adjacent VIN tissue. For the selected VIN cases, long‐term pathology follow up data were available from PALGA, the nationwide pathology registry, to evaluate for progression to VSCC.^4^ In total, formalin‐fixed, paraffin‐embedded (FFPE) tissue samples of 65 vulvar lesions yielded sufficient and adequate DNA amounts for laboratory analyses. These included 24 VSCC and 41 high‐grade VIN, including 10 pairs of VIN‐VSCC (ie meaning that both VIN and VSCC were from the same woman). Of the 41 VIN, 14 VIN were of women who did not develop VSCC during follow‐up (hereafter referred to as VIN^noVSCC^) and 27 VIN were of women with associated VSCC (hereafter referred to as VIN^VSCC^). In the VIN^noVSCC^ group, the follow‐up period in which no VSCC developed was at least 8.3 years (range 8.3‐14.8 years). In the VIN^VSCC^ group, 13/16 cases presented with both VIN and VSCC and 3 cases developed VSCC during follow‐up (range 1.5‐4.2 years). Collection, storage and use of archival tissue were performed in compliance with the “Code for Proper Secondary Use of Human Tissue in the Netherlands” (https://www.federa.org).

### 2.2 DNA isolation

DNA from VIN lesions was isolated following dissection of dysplastic regions (>70% dysplastic cells). For VSCC, whole tissue sections were used (>70% dysplastic cells). Laser capture micro‐dissection was performed using the Leica LMD6500 (Leica Microsystems, Wetzlar, Germany) as described before,^22^ with a few modifications.^23^ Eight micrometer thick sections were cut and mounted on PEN MembraneSlides (Leica). Sections were hydrated, stained with Mayer's Hematoxylin and completely dehydrated. Genomic DNA of micro‐dissected tissue was extracted after a 5‐day incubation period with lysis buffer (ATL buffer; QIAamp DNA microkit [Qiagen, Hilden, Germany]) and daily freshly added proteinase K (200 ng).^23^ For macrodissected VIN lesions and VSCC, DNA was isolated using the QIAamp DNA FFPE tissue kit (Qiagen). Concentration and purity was measured using the Qubit dsDNA BR assay kit (Thermo Fisher Scientific, Waltham, MA, USA) on the Qubit 2.0 Fluorometer (Thermo Fisher Scientific).

### 2.3 P16^INK4a^ immunohistochemistry and HPV testing

Determination of HPV‐status was performed using immunohistochemical staining of p16^INK4a^ followed by HPV DNA‐testing for p16^INK4a^‐positive samples, a test algorithm previously defined for the GP5+/6+ PCR test, and validated for HPV detection in FFPE tumor specimens.^24^ Immunohistochemical staining of p16^INK4a^ was performed on 3‐4 μm thick sections of FFPE specimens. Slides were considered positive when the lesion showed diffuse staining for p16^INK4a^.

Human papillomavirus DNA‐testing including detection of all HR‐HPV types and typing for HPV 16 and HPV 18 was performed using HPV risk assay^25^ (Self‐Screen B.V., Amsterdam, The Netherlands) according to the manufacturer's instructions. Only samples testing positive in PCR were considered true HPV‐positives.

### 2.4 Whole genome shallow‐sequencing

Two hundred and fifty nanogram DNA was fragmented by sonication (Covaris™ S2, Woburn, MA, USA). Samples were prepared using the TruSeq nano‐kit (Illumina San Diego, CA, USA) according to the manufacturer's instructions and sequenced on the Illumina HiSeq 2000 (Illumina San Diego) using the 50 bp single read (SR50) modus. Up to 24 samples were multiplexed on a single lane to yield ~8 million reads per sample.^26^

### 2.5 Sequencing analysis

Sequencing results were analyzed as described before.^26^ In short, we used the Bioconductor script QDNASeq.^26^ Mapping to the human reference genome (GRCh37/hg19) was done using the Burrow's Wheeler Alignmer (BWA).^27^ PCR duplicates and reads with a mapping quality lower than 37 were filtered out. Read counts were quantified in nonoverlapping 30 kb windows, followed by a simultaneous loess correction for sequence mappability and GC content. Problematic genome regions were filtered using a blacklist which was based on the 1000 Genome Project and the ENCODE project.^26^, ^28^ The X‐chromosome was excluded from analysis. Sequencing data was uploaded to ArrayExpress (Annotare; accession number E‐MTAB‐6113).

### 2.6 Data processing and clustering

Segmentation (α = .00000000001, number of SD's between means = 2) and subsequent calling of gained, amplified and lost regions was done using CGHcall. Segments with a probability score of ≥0.5 were considered gained, amplified or lost.^29^ CGHregions^30^ was used to reduce the dimension of the dataset, using thresholds of 0.1 (for CGHtest analysis, see below) or 0.01 (for all other analyses). The use of regions improves the effectiveness of the subsequent statistical analyses and facilitates the interpretation of the results.^30^

Unsupervised hierarchical clustering of different subsets of vulvar neoplasms was performed using a modified version of Weighted Clustering of Called Array CGH data (WECCA).^31^ The dendrograms were built using total linkage, which resulted in compact, well‐separated clusters.^30^ The distance between 2 features was defined as the symmetric Kullback‐Leibler divergence.^32^

### 2.7 Statistical analysis

For statistical analysis IBM SPSS Statistics (version 22.0.0.0; IBM, New York, NY, USA) and *R* (version 3.2.5) were used. The average total number of altered regions was compared between subgroups using the nonparametric Mann‐Whitney *U* test. To compare alterations between subgroups, a chi‐square‐test was performed using CGHtest,^23^ including a permutation‐based false discovery rate (FDR) correction for multiple testing. Alterations occurring <5% were a priori excluded and a *P*‐value <.05 with an FDR <0.2 was considered statistically significant.^33^ For comparisons between VIN and VSCC, paired cases were excluded from these analyses. Two‐sided *P*‐values below .05 were considered statistically significant.

## 3 RESULTS

### 3.1 HPV‐status in vulvar lesions

A series of 65 vulvar lesions, including 24 VSCC and 41 VIN with and without VSCC during follow‐up (referred to as VIN^VSCC^ and VIN^noVSCC^, respectively) were analyzed for HPV‐status using p16^INK4a^‐immunohistochemistry followed by detection of HR‐HPV DNA by PCR. This resulted in a series of 13 HPV‐negative VSCC, 11 HPV‐positive VSCC, 11 HPV‐negative VIN and 30 HPV‐positive VIN. All VIN^noVSCC^ (n = 14) were HPV‐positive. A total of 35 patients had HPV‐related lesions, 24 of these were HPV16‐positive, none were HPV18‐positive, and 11 were positive for other high‐risk HPV‐types.

### 3.2 Copy number aberrations in VSCC and VIN

#### 3.2.1 Frequencies of copy number aberrations

**Figure 1:**
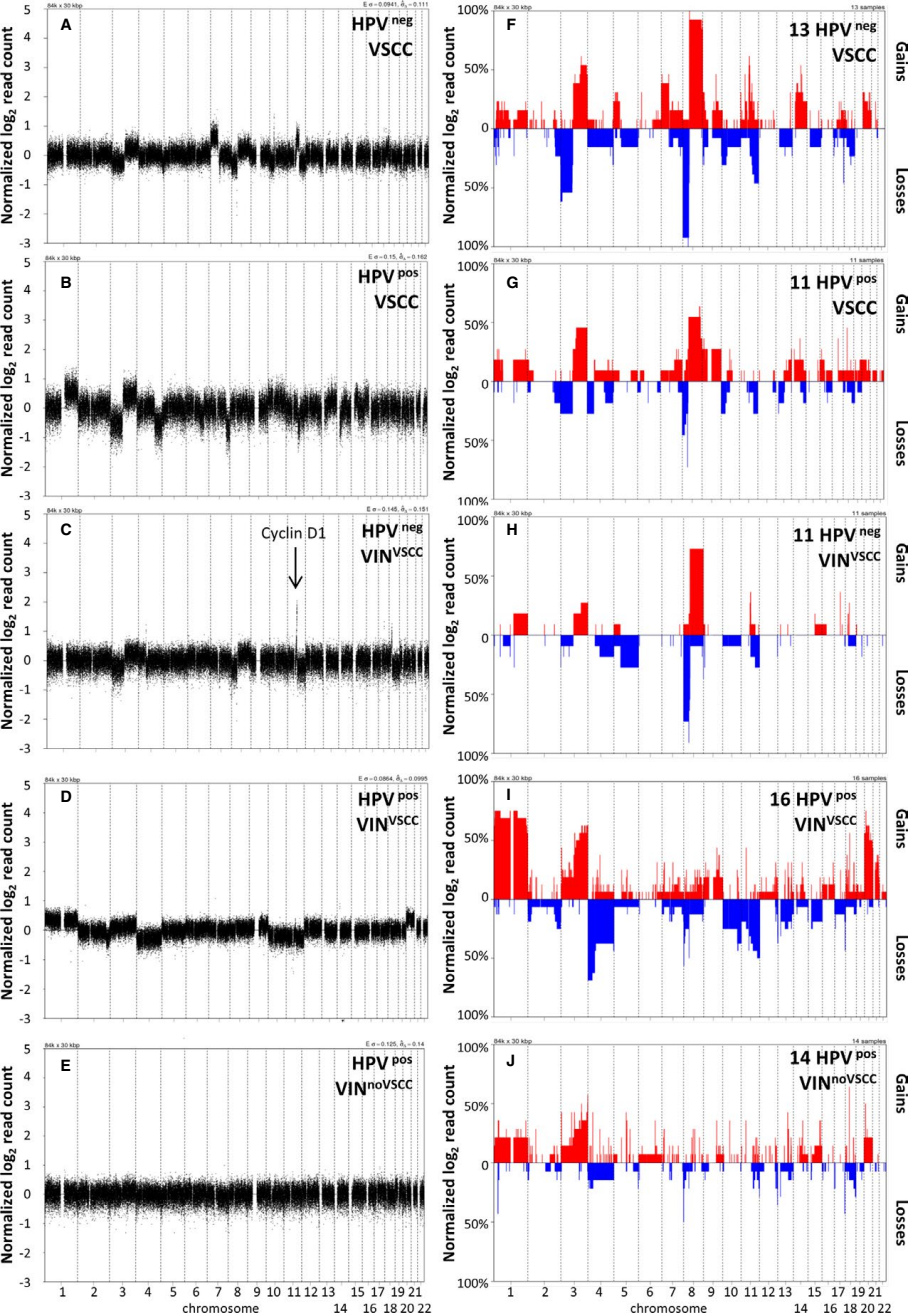
Copy number profiles and frequency plots of copy number alterations in vulvar squamous cell carcinomas (VSCC) and vulvar intraepithelial neoplasias (VIN). Representative copy number profiles are shown for (A) HPV‐negative VSCC, (B) HPV‐positive VSCC, (C) HPV‐negative VIN^VSCC^, (D) HPV‐positive VIN^VSCC^, and (E) HPV‐positive VIN ^no^ ^VSCC^. The arrow in (C) indicates 11q13/cyclin D1 amplification in this sample. Frequency plots are shown for (F) HPV‐negative VSCC, (G) HPV‐positive VSCC, (H) HPV‐negative VIN^VSCC^, (I) HPV‐positive VIN^VSCC^, and (J) HPV‐positive VIN ^no^ ^VSCC^. Gains are indicated in red (upper panel) and losses are indicated in blue (lower panel). Figures were generated using QDNAseq and CGHcall (see methods)

To analyze CNA, all samples were subjected to whole‐genome shallow‐sequencing. A representative selection of sequencing profiles is provided in Figure 1A‐E. HPV‐negative and ‐positive VSCC revealed a median percentage of altered 30 kb bins of 19.2% and 13.5% per tumor, respectively (Table 1). HPV‐negative VIN^VSCC^ had on average less alterations (8.0%) than HPV‐negative VSCC (19.2%, *P *=* *.009; Table 1). In contrast, no significantly different CNA frequency between HPV‐positive VIN^VSCC^ and HPV‐positive VSCC was found (ie 19.7% and 13.5%, respectively; *P *=* *.29). Interestingly, HPV‐positive VIN^noVSCC^ displayed significantly less chromosomal alterations than VIN^VSCC^ (8.0%, *P *=* *.022; Table 1).

**Table 1:** Percentage of 30 kb bins showing copy number alterations in different groups of VSCC and VIN

	% Total			% Losses			% Gains		
	Median	1st Qu	3rd Qu	Median	1st Qu	3rd Qu	Median	1st Qu	3rd Qu
HPV‐negative									
VSCC	19.2%	13.2%	33.0%	9.6%	4.9%	17.3%	11.1%	8.3%	16.2%
VIN^VSCC^	8.0%	5.4%	16.8%	3.9%	1.5%	9.0%	5.0%	2.1%	7.5%
HPV‐positive									
VSCC	13.5%	10.4%	21.8%	4.7%	0.3%	5.3%	10.6%	6.0%	15.2%
VIN^VSCC^	19.7%	11.5%	31.7%	6.0%	2.0%	14.8%	13.5%	7.9%	18.7%
VIN^noVSCC^	8.0%	2.7%	13.9%	2.1%	0.0%	6.6%	3.6%	0.5%	8.2%

Qu, quartile; VIN, vulvar intraepithelial neoplasia; VSCC, vulvar squamous cell carcinoma

To determine the affected chromosomal regions, frequency plots were made of chromosomal gains and losses for each group (Figure 1F‐J). All alterations occurring in at least 33% of each patient group are displayed in Table S1. Among these, gains were clearly overrepresented.

#### 3.2.2 Comparison of HPV‐positive VSCC with HPV‐negative VSCC

From Figure 1F‐G it becomes clear that the profiles of HPV‐positive and HPV‐negative VSCC are overlapping, both having frequent alterations of chromosome 3 and 8. However, the frequencies of these alterations are higher in the HPV‐negative VSCC. Almost all HPV‐negative VSCC showed chromosome 8 alterations, with gain of chromosome 8q11.21 (including the *SNAI2* gene) being detected in 100% of cases. In particular chromosome 3 and 8 isochromosome formation (3p/8p loss simultaneously with 3q/8q gain) was very common in HPV‐negative VSCC (54% chromosome 3p loss together with 3q gain, 85% chromosome 8p loss together with 8q gain), but also detected in a subset of HPV‐positive VSCC (45% for chromosome 3p loss together with 3q gain, 55% for chromosome 8p loss together with 8q gain). Comparison of HPV‐negative and ‐positive VSCC revealed 4 regions on chromosome 8p, of which 3 consecutive, that were significantly more frequent in HPV‐negative VSCC (Table S2). Additional recurrent alterations in HPV‐negative VSCC included gains at, amongst others, 7p, 11q12.3‐q14.2, and 14q13.3‐q24.1, and losses at 11q14.3‐q25 and 17q25.2‐q25.3 (Figure 1F; Table S1). Recurrent alterations in HPV‐positive VSCC, although occurring at much lower frequency than for HPV‐negative VSCC, comprised gains at 3q, 7q31.2‐q32.2, 8q11.21‐q24.22, 9p23‐22.3, 14q34, and 18q12.1, and losses at 3p26.3‐p26.1 and 8p23.3‐p21.2 (Figure 1G; Table S1).

#### 3.2.3 Comparison of HPV‐positive VIN^VSCC^ with HPV‐negative VIN^VSCC^

When comparing HPV‐positive VIN^VSCC^ with HPV‐negative VIN^VSCC^, multiple significant differences were found (Figure 1H‐I; Table S3). These included more frequent gains at several regions of chromosome 1p and 1q, 11p13 and 20p13‐q13.33 in HPV‐positive VIN^VSCC^. HPV‐negative VIN^VSCC^ on the other hand displayed significantly more gains at 8q and losses at 1p, 3p, and 8p.

#### 3.2.4 Comparison of HPV‐negative VIN^VSCC^ with HPV‐negative VSCC

Human papillomavirus‐negative VIN^VSCC^ displayed frequent gains of 8q as well as amplification of 11q13.3 and loss of 8p (Figure 1H; Table S1). Most of these alterations were also detected in HPV‐negative VSCC (Figure 1F). Only loss of 1p36.13 was statistically significantly more frequent in HPV‐negative VSCC (Table S4; excluding paired cases).

#### 3.2.5 Comparison of HPV‐positive VIN^VSCC^ with HPV‐positive VSCC

Human papillomavirus‐positive VIN^VSCC^ displayed, amongst others, frequent gains at chromosome 1p36.33‐p35.3, 1p35.2‐q44, 3q, 18q12.1, and 20pq, and losses at 4pq, 8p23.3, 10q25.1‐q26.3, and 11q13.4‐q25 (Figure 1I; Table S1). Interestingly, none of the HPV‐positive VSCC displayed a chromosome 1pq gain or 10q loss. Gains at chromosome 1 in VSCC involved different regions at 1p or 1q, but never gain of 1p and 1q combined. Also chromosome 20 gain, chromosome 4 loss and other frequent alterations were more common in HPV‐positive VIN^VSCC^ than in VSCC (Figure 1G,I). Gain of 1p34.3‐q21.1 was the only significantly differentially altered region between HPV‐positive VIN^VSCC^ and VSCC (Table S5; excluding paired cases).

When comparing paired HPV‐positive VIN‐VSCC cases, no significantly differently altered regions could be identified.

### 3.3 Copy number alterations in HPV‐positive VIN in relation to VSCC progression risk

**Figure 2:**
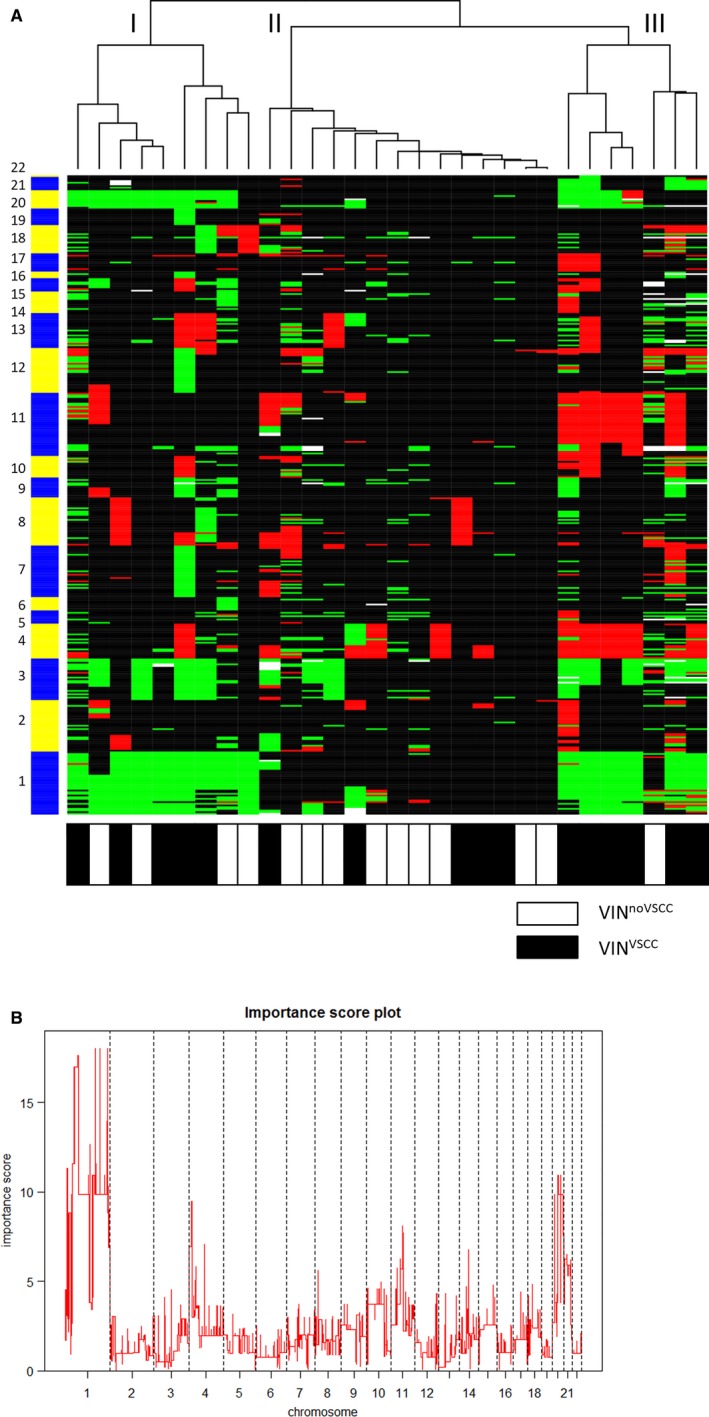
Clustering of HPV‐positive vulvar intraepithelial neoplasias (VIN). A, Weighted Clustering of Called Array CGH data (WECCA) output including a dendrogram, showing gains (green), and losses (red). Alternating chromosomes are indicated on the left. The panel below indicates the corresponding sample types. Three main clusters can be discerned. B, Importance score plot comparing vulvar intraepithelial neoplasia (VIN) lesions in the 3 clusters. For each chromosomal region the maximum pair‐wise symmetrized Kullback‐Leibler divergence was determined, revealing gain of chromosome 1pq as the most striking difference of the VIN lesions in the 3 clusters

To determine whether the cancer risk of VIN is reflected by CNA, we compared HPV‐positive VIN^noVSCC^ with HPV‐positive VIN^VSCC^. Unsupervised hierarchical clustering of all HPV‐positive VIN (either with or without VSCC) resulted in 3 clusters (Figure 2A). Cluster I contained both VIN^VSCC^ (5/9, 56%) and VIN^noVSCC^ (4/9, 44%) and is particularly characterized by gains at chromosomes 1, 3, and 20. Cluster II showing the least alterations contained more VIN^noVSCC^ (9/14, 64%) than VIN^VSCC^ (5/14, 36%). Cluster III is characterized by gains at chromosome 1, 3 and 20, similar to cluster I, as well as losses at chromosome 4 and 11. Cluster III included mostly VIN^VSCC^ (6/7, 86%) and only one VIN^noVSCC^ (1/7, 14%). The major differences in chromosomal alterations occurring in VIN lesions of the 3 clusters were determined by the maximum pairwise symmetrized Kullback‐Leibler divergence. Figure 2B shows the importance scores per chromosomal region, with a higher score indicating a larger contribution of the related alteration to the clustering result. This revealed that gain of chromosome 1pq was the most discriminatory alteration between the clusters, with some additional effect of chromosome 4p and chromosome 20. In line with this observation, especially a gain of 1pq was more frequent in HPV‐positive VIN^VSCC^ (81%), compared with VIN^noVSCC^ (21%, *P *=* *.001; Figure 2A). This gain of 1pq was not present in HPV‐positive VSCC, even not when the VIN and VSCC part were from the same patient (Figure S1).

Human papillomavirus‐positive VIN^noVSCC^ displayed frequent gains at several regions on chromosome 1pq, and 3pq, as well as gains at 4p14, 5q14.3, 5q15, 6q22.31, 10q21.1, 13q22.1, 18q12.1, and 20p12.2. Losses were most frequent at chromosome 1p35.3‐p35.2 and 17q25.2‐q25.3 (Figure 1J; Table S1). As described above, most of these were also affected in HPV‐positive VIN^VSCC^, though at different frequencies (Figure 1I; Table S1). The smallest regions of interest significantly associated with HPV‐positive VIN^VSCC^ as compared with VIN^noVSCC^ were gains of 1q21.2‐q44 and losses of 11p15.5‐p15.3, 11p15.3‐p13, and 11q14.1‐q21 (Table 2).

**Table 2:** Significantly altered regions between HPV‐positive VIN without and with VSCC

Region	Cytoband	*P*‐value	FDR	VIN^noVSCC^	VIN^VSCC^
(A) Gains					
chr1:149850001‐249180001	1q21.2‐q44	.0077	0.051	14%	63%
(B) Losses					
chr1:33960001‐35160001	1p35.1‐p34.3	.0126	0.0768	50%	6%
chr11:210001‐13470001^a^	11p15.5‐p15.3	.0179	0.0768	0	38%
chr11:13500001‐34560001^a^	11p15.3‐p13	.0179	0.0768	0	38%
chr11:82530001‐96120001	11q14.1‐q21	.0385	0.1311	7%	44%

FDR, false discovery rate; VIN, vulvar intraepithelial neoplasia; VSCC, vulvar squamous cell carcinoma. Gray shades indicate whether the regions are found more frequently in VIN^noVSCC^ or VIN^VSCC^, respectively.

a These regions are consecutive

### 3.4 Focal copy number alterations in vulvar lesions

Whole‐genome shallow sequencing also enabled the detection of focal aberrations, defined as <3 Mb^34^ at several genes (Table 3). Most notably, focal gains and amplifications of the regions encompassing *BCL2*,* CD44* (Figure S2A), 11q13/*CCND1* (Figure 1C, Figure S2B), desmocollins/desmogleins (*DSC*/*DSG*; Figure S2C), *JAG1*,* MET*, and *TP63* were found (>33% of HPV‐positive and/or ‐negative cases; Table 3). We observed that individual samples either displayed many focal alterations or very few, and that, consequently, many genes were co‐gained with other genes. Cyclin D1 amplifications were strongly associated with HPV‐negative lesions and absent in HPV‐positive lesions (*P *<* *.001). *BCL2* (*P *=* *.007) and *TP63* (*P = *.039) gains were more frequent in HPV‐positive lesions. Focal deletions were found at *RBFOX1/3* and *PTPRD*, the latter mainly occurring in HPV‐negative lesions (Figure S2D), an event that has been reported previously.^21^

**Table 3:** Selection of specific focal copy number alterations and high level amplifications and deletions (<3 Mb)

Gene	Chromosomal location	HPV‐status	VIN^noVSCC^	VIN^VSCC^	VSCC	Total (%)
TP63	3q28	+	9/14	10/16	5/11	59%
		−		1/11	6/13	29%
EGFR	7p11.2	+	5/14	2/16	1/11	20%
		−		0/11	6/13	25%
HIF1A	14q23.2	+	3/14	5/16	2/11	24%
		−		0/11	5/13	21%
JAG1	20p12.2	+	9/14	8/16	1/11	44%
		−		1/11	7/13	33%
CD44	11p13	+	7/14	10/16	6/11	56%
		−		3/11	7/13	42%
CCND1	11q13.3	+	0/14	0/16	0/11	0%
		−		5/11	6/13	46%
BCL2	18q21.33	+	7/14	8/16	4/11	46%
		−		0/11	3/13	13%
MET	7q31.2	+	6/14	8/16	4/11	44%
		−		0/11	6/13	25%
DSC/DSG	18q12.1	+	9/14	11/16	4/11	59%
		−		3/11	5/13	33%
PTPRD (del)	9p24.1‐p23	+	2/14	1/16	1/11	10%
		−		5/11	4/13	38%
RBFOX1 (del)	16p13.3	+	9/14	5/16	2/11	39%
		−		3/11	6/13	38%
RBFOX3 (del)	17q25.3	+	6/14	7/16	2/11	37%
		−		3/11	5/13	33%

del, deletion; VIN, vulvar intraepithelial neoplasia; VSCC, vulvar squamous cell carcinoma

## 4 DISCUSSION

**Figure 3:**
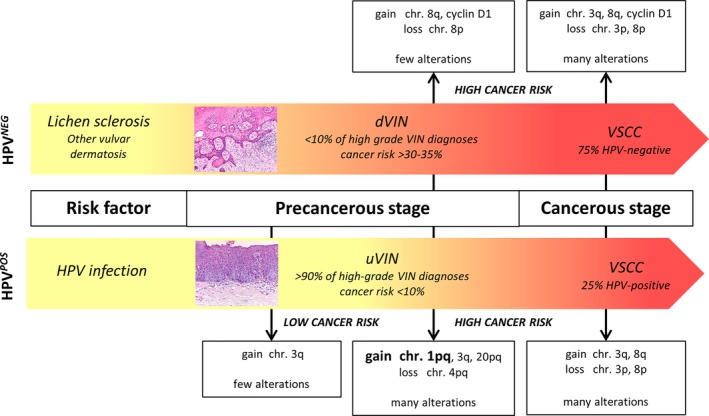
Schematic representation of HPV‐induced and HPV‐independent routes of vulvar carcinogenesis and the most frequently affected chromosomal regions. The involvement of specific copy number aberrations in the progression of vulvar intraepithelial neoplasia (VIN) towards vulvar squamous cell carcinoma (VSCC) is indicated for HPV‐negative (HPV^NEG^; upper panel) and HPV‐positive (HPV^POS^; lower panel) lesions. Chr., chromosome; dVIN, differentiated VIN; uVIN, usual VIN

Comprehensive chromosomal analysis of vulvar neoplastic lesions showed that HPV‐negative and HPV‐positive VSCC share many similarities, whereas DNA copy number profiles of VIN are more distinct (schematically presented in Figure 3). Most interestingly, the risk of VIN to progress into VSCC seems to be reflected by the extent of chromosomal alterations and specific CNA, in particular a gain of chromosome 1pq for HPV‐positive lesions.

Both HPV‐negative and HPV‐positive VSCC displayed frequent CNA at chromosome 3 and 8, with a highest frequency in HPV‐negative VSCC. These alterations are most likely of the isochromosome type since 3p/8p loss almost always occurred concurrently with 3q/8q gain in the same cases. Isochromosome formation of chromosome 8 has been reported previously for VSCC.^21^ Also a high frequency of chromosome 3 and 8 alterations in VSCC is in line with previous findings.^14^, ^17^, ^18^, ^20^, ^21^ Allen et al^18^ reported that 3q gain and 3p loss are more common in HPV‐positive VSCC and 8q gain in HPV‐negative VSCC. We found the same for 8q, but did not see major differences for chromosome 3 between HPV‐positive and ‐negative VSCC. Of interest, gains of chromosome 3q26‐q28 have been reported to be frequent in both HPV‐positive and ‐negative head and neck squamous cell carcinomas (HNSCC).^35^, ^36^, ^37^ Among putative target genes on chromosome 8 is the gene *SNAI2* (Slug), located on 8q11.21, which was gained in 100% of HPV‐negative VSCC. Slug is an epithelial‐mesenchymal transition promoting factor reported to be expressed in ~50% of VSCC, and primarily associated with HPV‐negative tumors.^38^ Loss of chromosome 11q affected both HPV‐positive and ‐negative VSCC in our study, as was also reported previously.^18^, ^20^

In contrast to the relatively similar CNA patterns in HPV‐positive and ‐negative VSCC, there were striking differences between their precursor lesions, with HPV‐negative VIN^VSCC^ showing similar CNA patterns as HPV‐negative VSCC (most notably chromosome 8 alterations), but HPV‐positive VIN^VSCC^ being very distinct. First, HPV‐positive VIN^VSCC^ displayed more alterations than HPV‐negative VIN^VSCC^. Second, the affected regions differed between HPV‐positive and ‐negative VIN^VSCC^. HPV‐positive VIN^VSCC^ frequently showed chromosome 1 gains (discussed below), chromosome 3q gain, chromosome 4 loss and chromosome 20 gain. In agreement with this, 2 previous studies on HPV‐positive VIN lesions demonstrated frequent gains at chromosome 1pq, 3q, 19, and/or 20q.^15^, ^16^ Gains of chromosome 1pq, 3q, and 20q were also common in HPV‐positive anal intraepithelial neoplasia.^39^ The frequent gains of chromosome 1 and 20 and loss of chromosome 4 mentioned above were rarely present in HPV‐positive VSCC.

Both VIN and VSCC displayed several focal alterations of well‐known cancer‐related genes, including *TP63*,* CD44*, cyclin D1, and *BCL2*. Altered CD44 protein and *TP63* mRNA expression in vulvar lesions has been reported by others.^40^, ^41^, ^42^, ^43^ Cyclin D1 amplifications were strongly associated with HPV‐negative lesions, whereas *BCL2* and *TP63* were more frequently affected in HPV‐positive cases. Gains of *TP63* and cyclin D1 have also been described in HNSCC.^35^, ^36^ Cyclin D1 amplification was detected in almost half of HPV‐negative VIN and VSCC, compared to 22% of HPV‐negative VSCC in a previous study.^44^ On the protein level, elevated cyclin D1 expression has been reported in 21%‐83% of VSCC, with p16^INK4a^‐negative VSCC being more frequently cyclin D1 positive than p16^INK4a^‐positive cases.^45^, ^46^ The absence of 11q13 amplifications in HPV‐positive VSCC as well as other HPV‐related tumors^37^ relates to a disruption of normal pRb function by the viral E7 protein, thereby obviating the need for cyclin D1 activation.

Most importantly, this is the first study in which HPV‐positive VIN^VSCC^ and VIN^noVSCC^ have been compared to identify potential copy number aberrations associated with cancer risk. Unsupervised hierarchical clustering analysis showed that these HPV‐positive VIN lesions displayed distinct copy number profiles. Particularly, gain of the complete chromosome 1 was significantly associated with the development of VSCC. Similar to our observation, Bryndorf et al^16^ and Purdie et al^15^ also found that HPV‐positive VIN lesions frequently exhibited gains of chromosome 1pq, whereas this was not the case for carcinomas. Based on this observation it has been suggested that these VIN lesions are unlikely to represent direct precursors of VSCC.^16^ However, our study showed that 1pq gain was present in most (13/16, 81%) HPV‐positive VIN^VSCC^, but infrequent (3/14, 21%), in VIN^noVSCC^. Interestingly, of the 3 VIN^noVSCC^ cases with 1pq gain, 2 were radically resected indicating that the aggressive treatment might have prevented progression of these lesions into cancer. Of the 11 VIN^noVSCC^ cases without 1pq gain, only 2 were radically resected indicating a low cancer risk in the remaining 9 lesions. Given the relatively low proportion of women with VIN that develop VSCC (ie 3%‐16%), many women with HPV‐positive VIN presumably have a low cancer‐risk profile and will therefore benefit from more conservative, for example, noninvasive management, whereas women with a high cancer‐risk profile should be approached more aggressively. Testing for chromosome 1 gain in HPV‐positive VIN, for example by fluorescent in situ hybridization, might help to identify VIN at risk for cancer progression and affected women may benefit from a more extensive treatment.

While HPV‐positive VIN^VSCC^ showed frequent 1pq gain, none of the HPV‐negative VIN showed gain of 1p and only 2 cases had gains of 1q. This may implicate a causative role for HPV‐infection in the gain of this specific region. Indeed, gains of chromosome 1 are also frequent in HPV‐positive anal and cervical intraepithelial neoplasia, and cervical carcinomas.^12^, ^32^, ^39^, ^47^ On the other hand, gains of chromosome 1 are infrequent in HPV‐positive head and neck squamous cell carcinomas,^32^, ^35^, ^37^, ^48^ as was the case for VSCC. An observation which is not completely understood is that in paired samples of adjacent HPV‐positive VIN and VSCC, a gain of 1pq was only detected in the VIN part and not the carcinoma. This might point to a mechanism of tumorigenesis in which 1pq gain enables the progression of VIN, but that upon invasive growth the copy numbers are normalized, potentially relating to intratumor heterogeneity. In addition to chromosome 1pq gain, also losses of 11p15‐p13 were significantly associated with VIN^VSCC^. Loss of 11p has been previously reported for both vulvar and cervical cancer,^19^ as well as anal intraepithelial neoplasia.^39^

In conclusion, we demonstrate a considerable heterogeneity of CNA in HPV‐positive and ‐negative VIN lesions, whereas HPV‐positive and ‐negative VSCC show more similar chromosomal profiles. Consistent with their more aggressive clinical course, HPV‐negative VIN show similar alterations as VSCC, affecting amongst others chromosome 3, 8, and 11q13.

We found clear differences between HPV‐positive VIN^VSCC^ and VIN^noVSCC^, with both an increased number of CNA and, most importantly, gain of chromosome 1 as a possible indicator of progression risk towards VSCC. These molecular distinctions may provide biomarkers that predict VSCC risk and would fulfill an urgent clinical need to improve current treatment modalities of women affected by VIN. To explore the clinical significance of our findings, further prospective studies are needed.

## CONFLICT OF INTEREST

DAMH, PJFS, RDMS and CJLMM are minority shareholders of Self‐screen B.V., a spin‐off company of VUmc; (2) Self‐screen B.V. holds patents related to the work (i.e., hrHPV test and methylation markers for cervical screening); (3) DAMH has been on the speaker’s bureau of Qiagen and serves occasionally on the scientific advisory boards of Pfizer and Bristol‐Meyers Squibb; (4) PJFS has been on the speakers bureau of Roche diagnostics, Gen‐Probe, Abbott, Qiagen and Seegene and has been a consultant for Crucell B.V.; (5) CJLMM has received speakers fee from GSK, Qiagen, SPMSD/Merck, Roche diagnostics, Menarini and Seegene, served occasionally on the scientific advisory board (expert meeting) of GSK, Qiagen, SPMSD/Merck, Roche and Genticel and has been by occasion consultant for Qiagen and Genticel; (6) CJLMM has small number of shares of Qiagen, was minority shareholder of Diassay B.V. until April 2016, and until 2014 he held a small number of certificates of shares in Delphi Biosciences, which went into receivership in 2014; (7) CJLMM is part‐time director of Self‐screen B.V. since September 2017; (8) The other authors declare no conflicts of interest.
