# Supplementary figures and images for: Molecular heterogeneity in human papillomavirus‐dependent and ‐independent vulvar carcinogenesis
Source: Cancer Med. 2018 Jul 20;7(9):4542–53. doi: 10.1002/cam4.1633 (PMC6144162; doi:10.1002/cam4.1633)

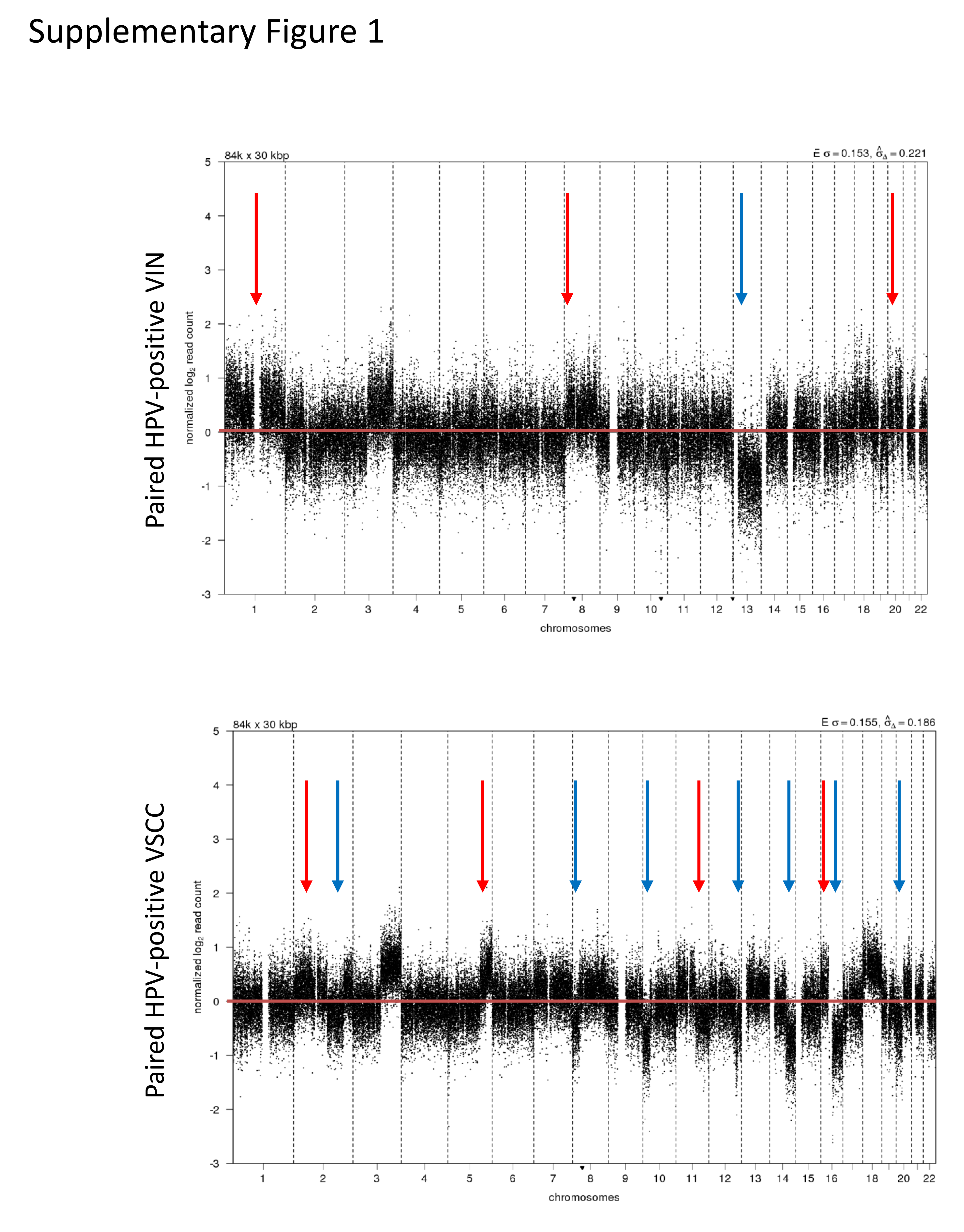

Supplement: Supplementary file 1 [file CAM4-7-4542-s001.tif]

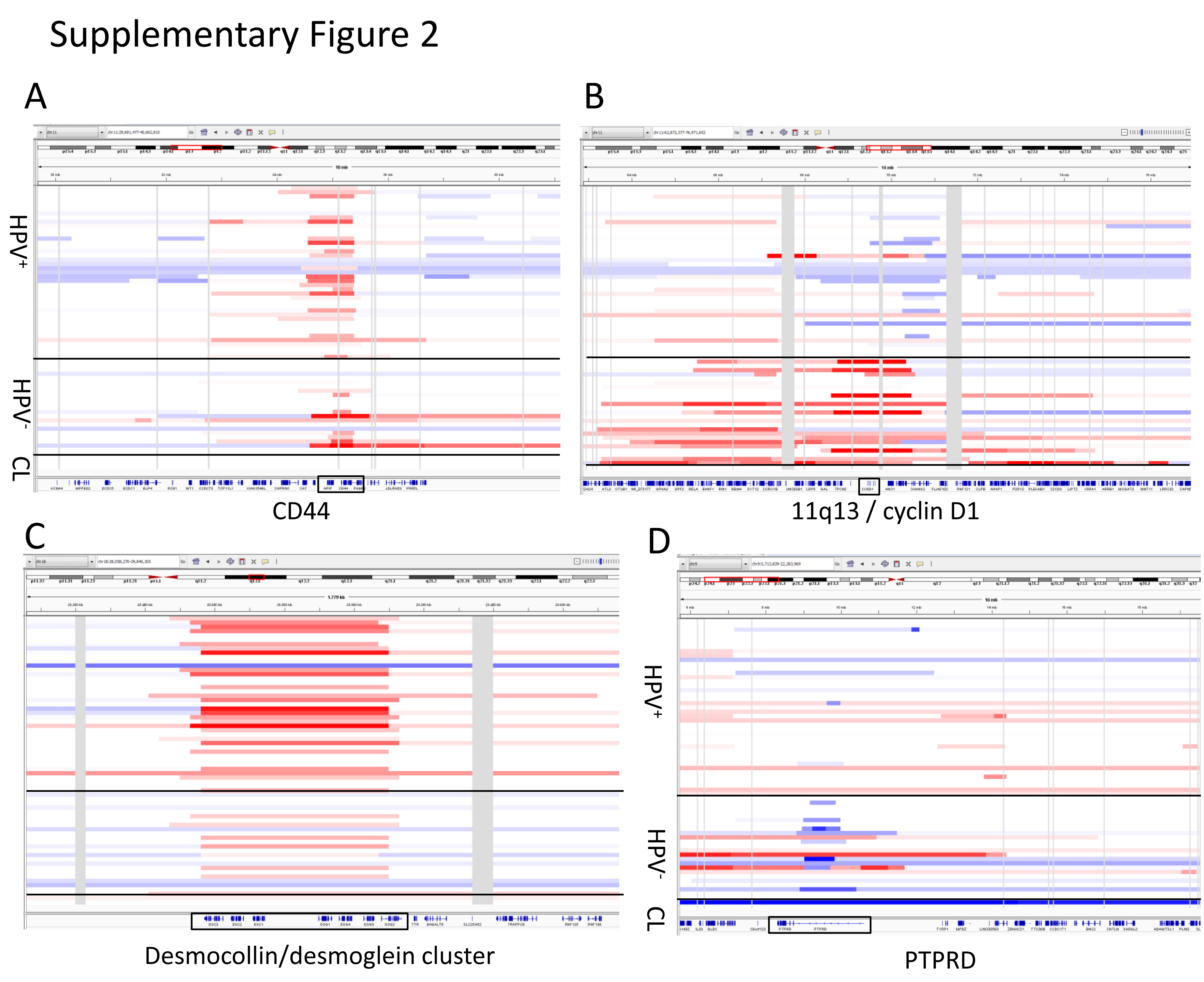

Supplement: Supplementary file 2 [file CAM4-7-4542-s002.tif]
