# Supplementary material for: Molecular heterogeneity in human papillomavirus‐dependent and ‐independent vulvar carcinogenesis
Source: Cancer Med. 2018 Jul 20;7(9):4542–53. doi: 10.1002/cam4.1633 (PMC6144162; doi:10.1002/cam4.1633)
Supplement: Supplementary file 3 [file CAM4-7-4542-s003.docx]

**Supplementary Figure Legends**

**Supplementary Figure 1. Example of a paired HPV-positive vulvar intraepithelial neoplasia (VIN) and vulvar squamous cell carcinoma (VSCC).**

The VIN lesion is shown in the upper panel and the VSCC in the lower panel. The two lesions both have shared alterations (losses of chromosome 2p and chromosome 4p) and unique gains (indicated with red arrows) and losses (indicated with blue arrows).

**Supplementary Figure 2. Examples of specific focal aberrations.**

(A-C) Integrative Genomics Viewer (version 2.3.75)^1^ screenshots of specific focal gains and amplifications of the genomic regions of (A) *CD44*, (B) 11q13/*CCND1*, and (C) the desmocollin/desmoglein cluster. Amplifications are shown in dark red and focal copy number gains in light red. (D) Integrative Genomics Viewer screenshots of specific focal deletion of the genomic region of *PTPRD*.

**References**

1. Robinson JT, Thorvaldsdottir H, Winckler W, Guttman M, Lander ES, Getz G, Mesirov JP. Integrative genomics viewer. *Nat Biotechnol* 2011;**29**: 24-6.
