# Supplementary material for: Molecular heterogeneity in human papillomavirus‐dependent and ‐independent vulvar carcinogenesis
Source: Cancer Med. 2018 Jul 20;7(9):4542–53. doi: 10.1002/cam4.1633 (PMC6144162; doi:10.1002/cam4.1633)
Supplement: Supplementary file 5 [file CAM4-7-4542-s005.docx]

**Supplementary Table 2-5**

| **Supplementary Table 2 - Significantly altered regions between HPV-negative and HPV-positive VSCC** | | | | | |
| --- | --- | --- | --- | --- | --- |
|  |  |  |  |  |  |
|  |  |  |  |  |  |
| **A) Gains** |  |  |  |  |  |
| **Region** | **Cytoband** | **P-value** | **FDR** | **HPV^-^ VSCC** | **HPV^+^ VSCC** |
| *none* |  |  |  |  |  |
|  |  |  |  |  |  |
| **B) Losses** |  |  |  |  |  |
| **Region** | **Cytoband** | **P-value** | **FDR** | **HPV^-^ VSCC** | **HPV^+^ VSCC** |
| chr8:180001-6240001 | 8p23.3-p23.2 | 0.0226 | 0.0496 | 92% | 45% |
| chr8:8100001-39210001* | 8p23.1-p11.22 | 0.0093 | 0.0204 | 92% | 36% |
| chr8:39240001-39360001* | 8p11.22 | 0.0073 | 0.0204 | 92% | 36% |
| chr8:39390001-43350001* | 8p11.22-p11.1 | 0.0006 | 0.003 | 92% | 18% |
|  |  |  |  |  |  |
| Abbreviations used: FDR, false discovery rate; HPV^-^, HPV-negative; HPV^+^, HPV-positive; VSCC, vulvar squamous cell carcinoma | | | | | |
|  |  |  |  |  |  |
| *These regions are consecutive | | | | | |

| **Supplementary Table 3 - Significantly altered regions between HPV-negative VIN and HPV-positive VIN with VSCC** | | | | | | |
| --- | --- | --- | --- | --- | --- | --- |
|  |  |  |  |  |  |  |
|  |  |  |  |  |  |  |
| **A) Gains** |  |  |  |  |  |  |
| **Region** | **Cytoband** | ***P*-value** | **FDR** | **HPV^-^ VIN** | **HPV^+^ VIN** |  |
| chr1:7770001-17940001 | 1p36.23-p36.13 | 0.0005 | 0.0007 | 0% | 69% |  |
| chr1:19200001-29580001 | 1p36.13-p35.3 | 0.0036 | 0.0048 | 9% | 69% |  |
| chr1:31080001-31110001* | 1p35.2 | 0.0417 | 0.0417 | 9% | 50% |  |
| chr1:31140001-33930001* | 1p35.2-p35.1 | 0.0174 | 0.0174 | 18% | 69% |  |
| chr1:35190001-36960001 | 1p34.3 | 0.022 | 0.022 | 27% | 75% |  |
| chr1:37890001-147360001 | 1p34.3-q21.1 | 0.0007 | 0.0011 | 0% | 63% |  |
| chr1:149040001-149190001 | 1q21.2 | 0.0401 | 0.0401 | 9% | 50% |  |
| chr1:149850001-249180001 | 1q21.2-q44 | 0.0478 | 0.0478 | 18% | 63% |  |
| chr8:47490001-48120001* | 8q11.21 | 0.0005 | 0.0007 | 64% | 0% |  |
| chr8:48150001-96270001* | 8q11.21-q22.1 | 0.0005 | 0.0007 | 73% | 6% |  |
| chr8:96300001-142290001 | 8q22.1-q24.3 | 0.0005 | 0.0007 | 73% | 6% |  |
| chr11:34590001-35370001 | 11p13 | 0.0174 | 0.0174 | 9% | 56% | *CD44 gene* |
| chr20:60001-62880001 | 20p13-q13.33 | 0.0025 | 0.0030 | 0% | 56% |  |
|  |  |  |  |  |  |  |
| **B) Losses** |  |  |  |  |  |  |
| **Region** | **Cytoband** | ***P*-value** | **FDR** | **HPV^-^ VIN** | **HPV^+^ VIN** |  |
| chr1:29610001-31050001* | 1p35.3-p35.2 | 0.0395 | 0.0896 | 64% | 19% |  |
| chr1:31080001-31110001* | 1p35.2 | 0.0079 | 0.0212 | 55% | 6% |  |
| chr3:90001-90180001 | 3p | 0.0253 | 0.0896 | 45% | 6% |  |
| chr8:6270001-6900001 | 8p23.2-p23.1 | 0.006 | 0.0181 | 82% | 25% |  |
| chr8:8100001-39210001* | 8p23.1-p11.22 | 0.0022 | 0.0093 | 82% | 19% |  |
| chr8:39240001-39360001* | 8p11.22 | 0.042 | 0.0896 | 91% | 50% |  |
| chr8:39390001-43350001* | 8p11.22-p11.1 | 0.0019 | 0.0093 | 82% | 19% |  |
|  |  |  |  |  |  |  |
| Abbreviation used: FDR, false discovery rate; HPV^-^, HPV-negative; HPV^+^, HPV-positive; VIN, vulvar intraepithelial neoplasia | | | | | | |
|  |  |  |  |  |  |  |

| **Supplementary Table 4 - Significantly altered regions between HPV-negative VIN with VSCC and HPV-negative VSCC*** | | | | | |
| --- | --- | --- | --- | --- | --- |
|  |  |  |  |  |  |
|  |  |  |  |  |  |
| **A) Gains** |  |  |  |  |  |
| **Region** | **Cytoband** | **P-value** | **FDR** | **VIN** | **VSCC** |
| *none* |  |  |  |  |  |
|  |  |  |  |  |  |
| **B) Losses** |  |  |  |  |  |
| **Region** | **Cytoband** | **P-value** | **FDR** | **VIN** | **VSCC** |
| chr1:17970001-19170001 | 1p36.13 | 0.0158 | 0.1135 | 13% | 80% |
|  |  |  |  |  |  |
| Abbreviations used: FDR, false discovery rate; VIN, vulvar intraepithelial neoplasia; VSCC, vulvar squamous cell carcinoma | | | | | |
|  |  |  |  |  |  |
| *Excluding paired cases | | | | | |

| **Supplementary Table 5 - Significantly altered regions between HPV-positive VIN with VSCC and HPV-positive VSCC*** | | | | | |
| --- | --- | --- | --- | --- | --- |
|  |  |  |  |  |  |
|  |  |  |  |  |  |
| **A) Gains** |  |  |  |  |  |
| **Region** | **Cytoband** | **P-value** | **FDR** | **VIN** | **VSCC** |
| chr1:37890001-147360001 | 1p34.3-q21.1 | 0.0153 | 0.1051 | 78% | 0% |
|  |  |  |  |  |  |
| **B) Losses** |  |  |  |  |  |
| **Region** | **Cytoband** | **P-value** | **FDR** | **VIN** | **VSCC** |
| *none* |  |  |  |  |  |
|  |  |  |  |  |  |
| Abbreviations used: FDR, false discovery rate; VIN, vulvar intraepithelial neoplasia; VSCC, vulvar squamous cell carcinoma | | | | | |
|  |  |  |  |  |  |
| *Excluding paired cases | | | | | |
